# JianPi JieDu Recipe Inhibits Epithelial-to-Mesenchymal Transition in Colorectal Cancer through TGF-*β*/Smad Mediated Snail/E-Cadherin Expression

**DOI:** 10.1155/2017/2613198

**Published:** 2017-02-16

**Authors:** Xuan Liu, Qing Ji, Wanli Deng, Ni Chai, Yuanyuan Feng, Lihong Zhou, Hua Sui, Chunpu Li, Xiaoting Sun, Qi Li

**Affiliations:** Department of Medical Oncology, Shuguang Hospital, Shanghai University of Traditional Chinese Medicine, 528 Zhangheng Road, Shanghai 201203, China

## Abstract

JPJD was an ideal alternative traditional Chinese medicine compound in the prevention and treatment of CRC, but its underlying mechanisms has not been fully elucidated. In this study, we demonstrated in vitro that TGF-*β*-induced EMT promoted the invasion and metastasis of CRC cells, reduced the expression of E-cadherin, and elevated the expression of Vimentin. However, JPJD could inhibit the invasive and migratory ability of TGF-*β*-stimulated CRC cells in a concentration-dependent manner through increasing the expression of E-cadherin and repressing the expression of Vimentin, as well as the inhibition of TGF-*β*/Smad signaling pathway. Meanwhile, JPJD reduced the transcriptional activities of EMT-associated factors Snail and E-cadherin during the initiation of TGF-*β*-induced EMT. In vivo, the results demonstrated that JPJD can significantly inhibit the liver and lung metastasis of orthotopic CRC tumor in nude mice, as well as significantly prolonging the survival time of tumor-bearing in a dose-dependent manner. Additionally, JPJD can upregulate the expression of E-cadherin and Smad2/3 in the cytoplasm and downregulate the expression of Vimentin, p-Smad2/3, and Snail in the orthotopic CRC tumor tissues. In conclusions, our new findings provided evidence that JPJD could inhibit TGF-*β*-induced EMT in CRC through TGF-*β*/Smad mediated Snail/E-cadherin expression.

## 1. Introduction

The incidence and mortality of colorectal cancer (CRC) are increasing year by year [1], and many CRC patients were found in advanced periods. The prognosis of advanced CRC patients is not ideal usually, even after surgery and chemotherapy treatment. Generally, the incidences of tumors including CRC, gastric cancer, liver cancer are mostly caused by the interaction of many tumor associated factors. For example, upregulation of oncogenes and downregulation of anti-oncogenes will make normal cells escape the defenses of immune systems, followed by the formation of tumors and further tissues invasion and metastases [2]. Epithelial-to-mesenchymal transition (EMT) often occurs in the progression of various epithelial cell carcinomas and is closely associated with invasion and metastasis of tumors including CRC [3]. Presently, many cytokines such as TGF-*β*, HGF, and EGF are generally used to induce EMT [4–6].

JianPi JieDu Recipe (JPJD) is a traditional Chinese medicine compound from clinical experience, with the function of tonifying Qi and spleen, eliminating dampness, and regulating Qi and detoxification, and it is mainly used for the prevention of gastrointestinal cancer recurrence and metastasis. JPJD was composed of Radix Astragal (Huangqi), Rhizoma Atractylodis Macrocephala (Baizhu), wild grapevines (Yeputaoteng), Fructus Akebia (Bayuezha),* Salvia chinensis* Benth. (Shijianchuan), and* Evodia rutaecarpa* (Wuzhuyu). Previous clinical research has demonstrated that JPJD has an excellent regulating effect in improving patients' symptoms and promoting quality of lives, and it was an ideal alternative traditional Chinese medicine compound in the prevention and treatment of the invasion and metastasis of CRC [7]. Numerous basic researches by our group have suggested the great potential of JPJD for further investigation [8, 9]. Nevertheless, the underlying mechanism of JPJD in preventing CRC recurrence and metastasis is not very clear. This study will aim to investigate the effect mechanism of JPJD on TGF-*β*-induced EMT in CRC LoVo cells and provide an experimental basis for targeted therapy of CRC.

## 2. Materials and Methods

### 2.1. Reagents and Materials

Matrigel was purchased from BD (USA). Fibronectin was purchased from Roche (USA). Transwell plates were purchased from Corning (USA). Recombinant human TGF-*β*1 was purchased from R&D (USA). The ethanol extracts of JPJD were extracted by Institute of Materia Medica affiliated Shanghai University of Traditional Chinese Medicine. The ethanol extracts of JPJD were dissolved at a concentration of 20 mg/ml as a stock solution in F12K medium containing 10% fetal bovine serum (FBS), followed by ultrasonic mixing overnight and filtration with 0.22 *μ*m filter. Rabbit monoclonal antibodies against human E-cadherin, Vimentin, Snail, Smad2/3, p-Smad2/3, MMP-2, MMP-9, *β*-actin, and PCNA were purchased from Cell Signaling Technology (USA).

### 2.2. CCK Assay for Cell Proliferation

Human CRC cell line LoVo (ATCC, USA) was maintained in F12K medium containing 10% fetal bovine serum (FBS), 100 U/mL penicillin, and 100 *μ*g/mL streptomycin and incubated in a humidified, 5% CO_2_ atmosphere, at 37°C. Briefly, LoVo cells were firstly seeded in 96-well plates at 1 × 10^4^ cells/well. When the cells reached 60% confluence, the medium was replaced with fresh medium containing different concentrations of JPJD. After incubating for 24 h, 48 h, and 72 h, the medium was discarded, and 100 *μ*l medium containing CCK-8 reagent was added. After incubating for another 4 h, the absorbance was determined at 450 nm using a microplate reader (Bio-Rad, USA). All the experiments were repeated at least three times.

### 2.3. Invasion and Migration Assay

The assay method was adopted from previous reports [8]. Briefly, LoVo cells (5 × 10^5^, in F12K medium with 0.5% FBS) pretreated with or without different concentration of JPJD were seeded into the upper part of a transwell chamber. For migration, 600 *μ*l F12K medium with 10 *μ*g/ml fibronectin and 15% FBS was added in the lower part of the chamber. For invasion, 100 *μ*l Matrigel was firstly added onto the upper transwell chamber before LoVo cells were seeded, and the following procedures were the same as migration assay. The migration and invasion assay were incubated for 24 h and 48 h, respectively. After that, the migrated or invasive cells were analyzed by crystal violet staining, and five random views were selected to count the migrated cells by a DMI3000 B inverted microscope at the magnification of 200x (Leica, Germany). Each experiment was repeated at least three times.

### 2.4. Western Blot

Cells were washed by Phosphate Buffered Saline (PBS) three times, and then the whole cell, cytoplasm, and nuclear proteins were extracted according to the procedures from ProteoJET Cytoplasmic Kit (Fermentas, USA). The extracted proteins were quantified and loaded for SDS-PAGE gel electrophoresis, transferred to PVDF membranes, and blocked in 10% milk. Next, the membranes were incubated with the primary antibodies and subsequently the HRP-conjugated secondary antibodies. All the resulting immunocomplexes were visualized by enhanced chemiluminescence, followed by directly photographing and quantitative analyzing (ChemiScope 3600, China).

### 2.5. RT-PCR

The gene sequences of Snail and E-cadherin were acquired from GenBank, and the primers were designed with Primer 5.0 software. All the primers were synthesized by Shanghai ShineGene Company. For real-time PCR, the primers were designed as follows: GAPDH, 5-GGTGGTCTCCTCTGACTTCAA-3 (forward) and 5-CCAAATTCGTTGTCATACCAG-3 (reverse); E-cadherin, 5-TAGAGGGTCACCGCGTC-3 (forward) and 5-GGGCTGGAGTCTGAACT-3 (reverse); Snail, 5-CAATCGGAAGCCTAACTA-3 (forward) and 5-CAGATGAGCATTGGCAGCG-3 (reverse); MMP-2, 5-AGACATACATCTTTGCTG-3 (forward) and 5-CTTGAAGAAGTAGCTGTG-3 (reverse); MMP-9, 5-CCTGGAGACCTGAGAACCAAT-3 (forward) and 5-GATTTCGACTCTCCACGCAT-3 (reverse). Real-time PCR was performed in Applied Biosystems 7300 System (Applied Biosystems Deutschland GmbH), according to the instructions of Premix Ex Taq Kit (Takara, Dalian, China). All assays were performed in triplicate and independently repeated three times.

### 2.6. Dual-Luciferase Assay for Promoter Activity

LoVo cells (1 × 10^5^) were firstly seeded in 6-well plates and incubated overnight in a humidified, 5% CO_2_ atmosphere, at 37°C. To test E-cadherin (CDH1) promoter activity, LoVo cells were cotransfected with either the recombinant plasmid pGL3-basic-CDH1-promoter or pGL3-basic-mut-CDH1-promoter, with a control positive plasmid pRL-SV40. After transfection for 24 h, the promoter activity was measured using a dual-luciferase assay kit (Beyotime Institute of Biotechnology, China) according to the manufacturer's instructions.

### 2.7. Orthotopic Transplantation Tumor Assay

100 *μ*l single-cell suspensions of LoVo cells (2 × 10^6^) were injected into the subcutaneous area of female BALB/c nude mice (4–6 weeks old) obtained from SLAC Laboratory (Shanghai, China). After the transplanted tumors reached 100 mm^3^, the tumors were excised, fractionated, and transplanted into the appendix of nude mice. After two weeks, the mice were randomized into four groups of 8 animals each. JPJDs with low-dose (250 mg/kg/day), middle-dose (500 mg/kg/day), and high-dose (1000 mg/kg/day) with control physiological saline were intragastrically administrated every day for the above four groups. After 28 days, the nude mice were sacrificed. The primary tumors, the lung, and liver of the mice were surgically removed and investigated by HE (hematoxylin-eosin) staining. All experimental protocols were reviewed and approved by the animal ethics committee of Shuguang Hospital, Shanghai University of Traditional Chinese Medicine.

### 2.8. ELISA

The medium supernatants were collected, centrifuged at 3000 rpm for 10 min, and measured for MMP-2 and MMP-9 concentration using an ELISA kit (Bogoo, Shanghai, China) according to manufacturer's instruction.

### 2.9. Statistical Analysis

All data were presented as means with standard deviation (SD), and the statistic comparison was performed using Student's *t*-test, with the significance level at *P* < 0.05. All statistical analyses were performed with SPSS 18.0 software.

## 3. Results

### 3.1. Inhibitory Effect of JPJD on EMT in CRC Cells

**Figure 1:**
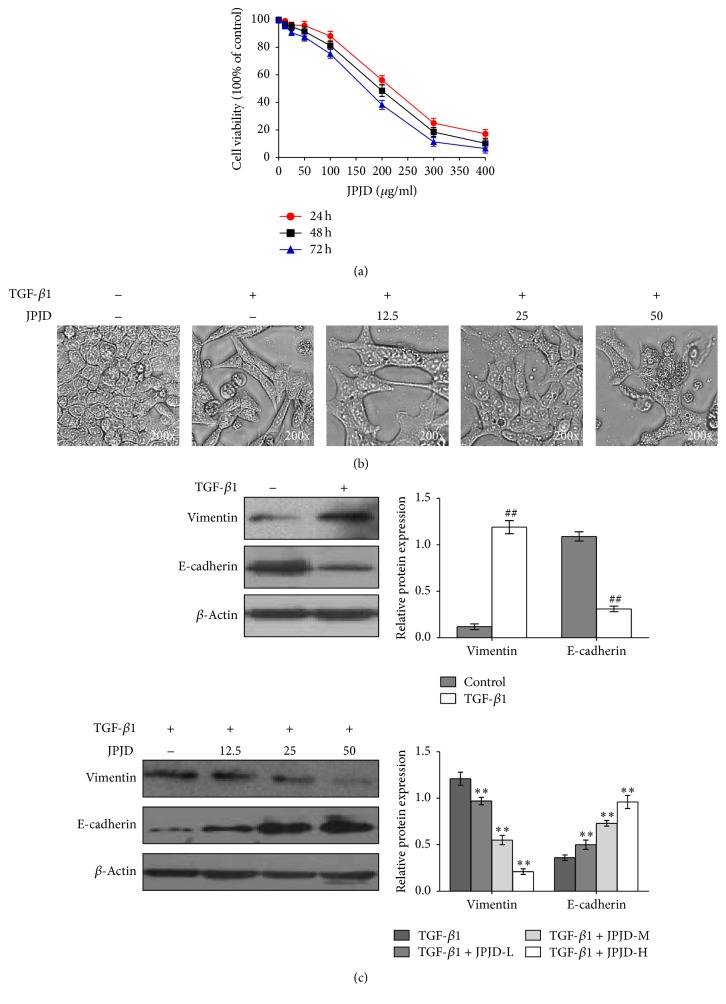
Inhibitory effect of JPJD on the EMT of TGF-*β*-stimulated LoVo cells. (a) Inhibitory effect of JPJD on the proliferation of LoVo cells. LoVo cells were treated with JPJD of different concentrations including 12.5 *μ*g/mL, 25 *μ*g/mL, 50 *μ*g/mL, 100 *μ*g/mL, 200 *μ*g/mL, 300 *μ*g/mL, and 400 *μ*g/mL. After 24 h, 48 h, and 72 h, CCK-8 assay was performed to investigate the growth inhibitory effect of JPJD on LoVo cells. (b) Morphological observation of the TGF-*β*-stimulated or JPJD treated LoVo cells. The concentration of TGF-*β* used in the whole research was 10 ng/ml. The concentrations of JPJDs were calculated according to CCK-8 results, including 12.5 *μ*g/mL, 25 *μ*g/mL, and 50 *μ*g/mL. The magnification of the microscopic pictures was ×200. (c) Impact of JPJD on the protein expression of Vimentin and E-cadherin in TGF-*β*-stimulated LoVo cells. The time of TGF-*β*-stimulation and JPJD treatment was 48 hours. ^##^*P* < 0.01, versus control LoVo cells, and ^*∗*^*P* < 0.05 and ^*∗∗*^*P* < 0.01, versus only TGF-*β*-stimulated cells.

CRC LoVo cells were treated with JPJD of different concentration including 12.5 *μ*g/mL, 25 *μ*g/mL, 50 *μ*g/mL, 100 *μ*g/mL, 200 *μ*g/mL, 300 *μ*g/mL, and 400 *μ*g/mL. After 24 h, 48 h, and 72 h, CCK-8 assay was performed to investigate the growth inhibitory effect of JPJD on LoVo cells. The results suggested that JPJD inhibited the proliferation of LoVo cells in a concentration- and time-dependent manner. The growth inhibition rate was about 10% after treatment with JPJD at 50 *μ*g/mL for 48 h (Figure 1(a)), indicating that the JPJD of less than 50 *μ*g/mL has little effect on the proliferation of LoVo cells. Therefore, in subsequent experiments, JPJDs of 12.5 *μ*g/mL, 25 *μ*g/mL, and 50 *μ*g/mL were selected as low, middle, and high concentration for further studies in vitro.

Many cytokines such as TGF-*β*, HGF, and EGF are used to induce EMT for a variety of epithelial cells in vitro [4–6], providing cell models for further mechanic and functional studies. Here, we firstly observed the effect of TGF-*β* cytokines on the morphology of CRC LoVo cells under a microscope. The results showed that, being treated with 10 ng/ml TGF-*β* for 48 h, the morphology of LoVo cells made significant changes, for example, being easier to form a fine spindle, and intercellular adhesion decreased remarkably (Figure 1(b)). Our previous experiments have found that JPJD could suppress the invasion and metastasis of LoVo cells, so whether JPJD could inhibit EMT of LoVo cells is what we need to focus on subsequently. From the morphology observation we found that JPJD shows excellent inhibitory effect on TGF-*β*-induced EMT in a concentration-dependent manner.

Numerous studies have shown that, in the process of EMT, the cells lose their epithelial phenotype accompanied by a reduction of E-cadherin expression and gradually gained mesenchymal phenotype along with an increase of Vimentin expression. Therefore, E-cadherin and Vimentin were subsequently detected by western blot. From the results we found, compared with the control LoVo cells without TGF-*β* treatment, the Vimentin expression elevated obviously in TGF-*β* treated LoVo cells, while the E-cadherin expression decreased remarkably (Figure 1(c), Supplementary Figure 1, in Supplementary Material available online at https://doi.org/10.1155/2017/2613198), suggesting the apparent EMT of LoVo cells. Further experiment demonstrated that, in LoVo cells pretreated with TGF-*β*, after treatment with JPJD of low concentration, middle concentration, and high concentration, the intracellular Vimentin expression decreased, while the E-cadherin expression showed a significant increase, in a concentration-dependent manner (Figure 1(c), Supplementary Figure 1). The results implied that JPJD may partially inhibit TGF-*β*-induced EMT in LoVo cells.

**Figure 2:**
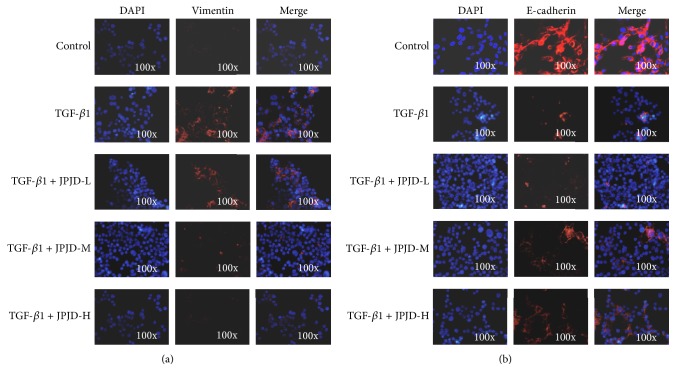
Effect of JPJD on the expression and location of Vimentin and E-cadherin in TGF-*β*-stimulated LoVo cells by immunofluorescence detection. (a) Effect of JPJD on the expression and location of Vimentin. (b) Effect of JPJD on the expression and location of E-cadherin. Here, JPJD-L is JPJD low concentration (12.5 *μ*g/mL), JPJD-M is JPJD middle concentration (25 *μ*g/mL), JPJD-H is JPJD high concentration (50 *μ*g/mL), and the time of TGF-*β*-stimulation and JPJD treatment was 48 hours. The magnification of the microscopic pictures was ×100.

Simultaneously, we also used immunofluorescence techniques to investigate the E-cadherin and Vimentin expression in LoVo cells, and the results were consistent with western blot. After TGF-*β* treatment in LoVo cells, the Vimentin expression elevated while the E-cadherin expression decreased remarkably (Figure 2).

### 3.2. Influence of JPJD on the Invasion and Metastasis of TGF-*β*-Stimulated CRC Cells

**Figure 3:**
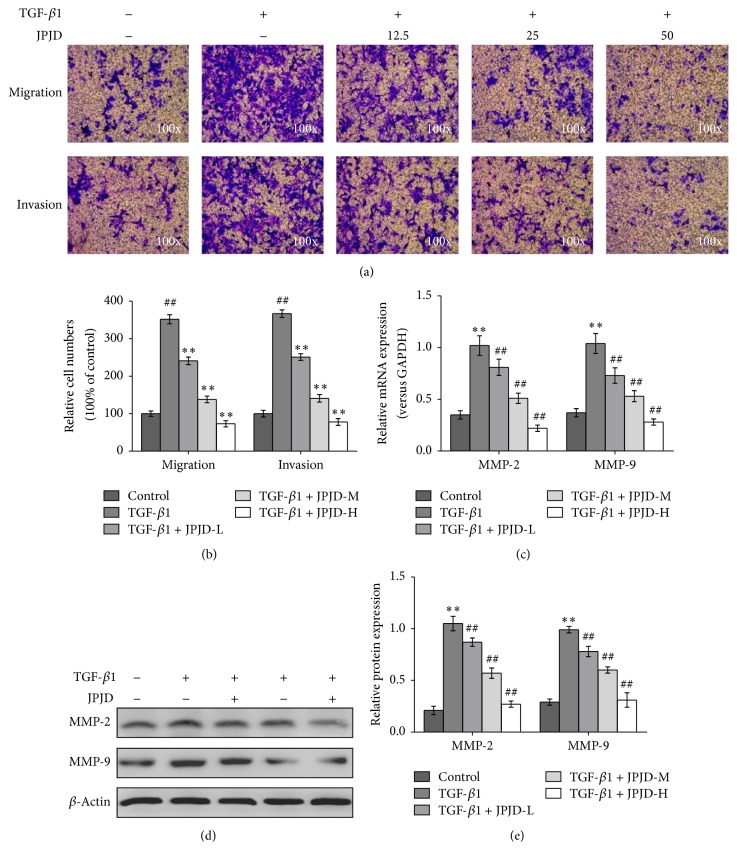
Impact of JPJD on the invasion and metastasis of TGF-*β*-stimulated LoVo cells. (a) Transwell experiment was performed to observe the impact of JPJD on the invasion and metastasis of TGF-*β*-stimulated LoVo cells. The magnification of the microscopic pictures was ×100. (b) Numbers of invasive and migrated cells shown as mean ± SD, *n* = 3. (c, d, e) Real-time PCR, western blot, and ELISA were, respectively, performed to test the effect of JPJD on the MMP-2 and MMP-9 expression of TGF-*β*-stimulated LoVo cells, as well as the secretory levels of MMP-2/MMP-9 in the culture medium of TGF-*β*-stimulated LoVo cells. Here, JPJD-L is 12.5 *μ*g/mL, JPJD-M is 25 *μ*g/mL, JPJD-H is 50 *μ*g/mL, and the time of TGF-*β*-stimulation and JPJD treatment was 48 hours. ^##^*P* < 0.01, versus control LoVo cells, and ^*∗*^*P* < 0.05 and ^*∗∗*^*P* < 0.01, versus only TGF-*β*-stimulated cells.

Since TGF-*β* factor could stimulate EMT process of LoVo cells, it was very necessary to further probe whether the function of cell invasion and metastasis would change. Transwell experiments demonstrated that, after being stimulated by TGF-*β* factor for 48 h, LoVo cells showed significantly increased ability to penetrate the basement membrane and Matrigel, compared with the control LoVo cells (Figure 3(a)), which suggested that TGF-*β* stimulation promoted the invasion and metastasis abilities of LoVo cells. Meanwhile, we observed the effect of JPJD on the invasion and metastasis of TGF-*β*-stimulated LoVo cells. The results showed that JPJD inhibited the invasion and metastasis of TGF-*β*-stimulated LoVo cells in a concentration-dependent manner (Figures 3(a) and 3(b)), suggesting that JPJD may inhibit TGF-*β*-induced EMT, thereby inhibiting the invasion and metastasis of LoVo cells.

In the pathological process of tumor invasion and metastasis, it is very easy for the extracellular matrix (ECM) to get damage. In the reported ECM enzymes, the matrix metalloproteinase (MMP) enzymes, a class of proteolytic enzymes, play a key role in the bioprocess, especially the MMP-2 and MMP-9. Real-time PCR and western blot results showed that TGF-*β* stimulation could significantly upregulate the mRNA and protein expressions of MMP-2 and MMP-9; however, in TGF-*β*-stimulated LoVo cells, JPJD could inhibit the mRNA and protein expressions of MMP-2 and MMP-9 in a concentration-dependent manner (Figures 3(c) and 3(d), Supplementary Figure 2). Further ELISA data also demonstrated that TGF-*β* stimulation could significantly upregulate the secreted levels of MMP-2 and MMP-9 protein in the culture medium of TGF-*β*-stimulated LoVo cells, while JPJD could inhibit the secreted levels of MMP-2 and MMP-9 in a concentration-dependent manner (Figure 3(e)).

### 3.3. JPJD Inhibited EMT in CRC through TGF-*β*/Smad Signaling Pathway

**Figure 4:**
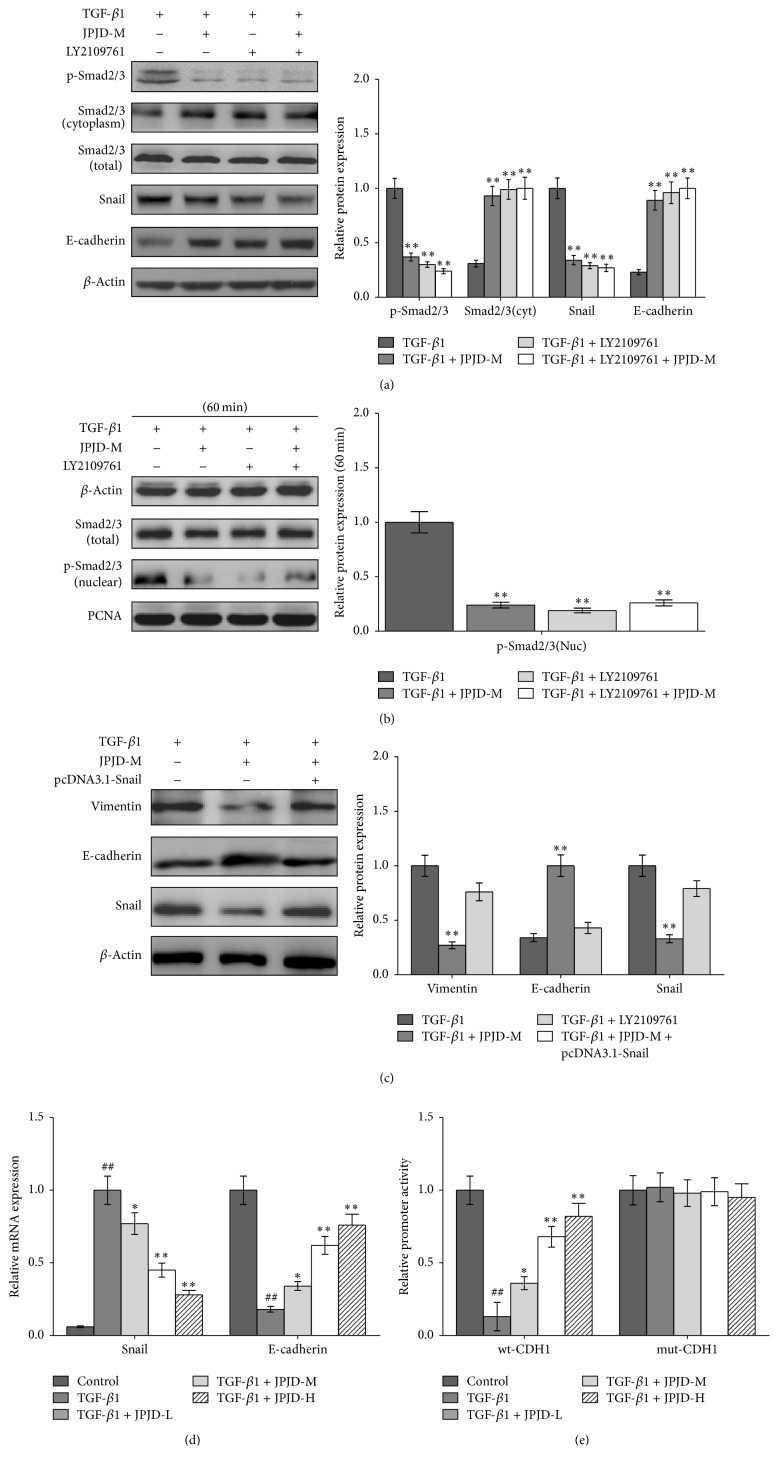
JPJD inhibited the EMT of CRC cells through TGF-*β*/Smad signaling pathway. (a) Western blot was applied to investigate the regulatory effect of JPJD on TGF-*β*/Smad signaling pathway. TGF-*β*-stimulated LoVo cells were treated with JPJD-M and/or TGF*β*RI/II inhibitor (LY2109761) for 48 hours. (b) Instantaneous inhibitory effect of JPJD on the activation of TGF-*β*/Smad signaling pathway. TGF-*β*-stimulated LoVo cells were treated with JPJD-M or LY2109761 only for 60 min or pretreated with LY2109761 followed by JPJD-M treatment for 60 min. (c) The process of EMT was detected followed by JPJD treatment and reexpression of Snail. (d) Real-time PCR was performed to measure the regulatory effect of JPJD on mRNA expression of E-cadherin and Snail. (e) Dual-luciferase assay for the inhibitory effect of JPJD on the promoter activity of CDH1 encoding E-cadherin Here, JPJD-L is 12.5 *μ*g/mL, JPJD-M is 25 *μ*g/mL, and JPJD-H is 50 *μ*g/mL. ^##^*P* < 0.01, versus control LoVo cells; ^*∗*^*P* < 0.05 and ^*∗∗*^*P* < 0.01, versus only TGF-*β*-stimulated LoVo cells.

Previous studies have demonstrated that JPJD has significantly inhibitory effect on TGF-*β*-stimulated EMT, but the detailed effect mechanism is unclear. Previous studies [10] have demonstrated that the TGF*β*RI/II inhibitor (LY2109761) could inhibit the phosphorylation of Smad2/3 induced by TGF*β*1 in LoVo cells. In the present study, we firstly examined the inhibitory activity of JPJD against TGF-*β*-induced Smad phosphorylation after 48 h of TGF-*β* stimulation. The data showed that JPJD could downregulate the levels of p-Smad2/3 and upregulate the cytoplasmic levels of Smad2/3 in TGF-*β*-stimulated LoVo cells, as well as the decreased expression of Snail protein and increased expression of E-cadherin protein, while the total expression of Smad2/3 changed little (Figure 4(a), Supplementary Figure 3). As is known to all that, JPJD can inhibit Smads signaling pathway, and TGF-*β* plays key regulatory role on Smads signaling pathway, and it is necessary to investigate whether JPJD could inhibit the EMT through TGF-*β* mediated Smads signaling pathway. To verify this hypothesis, TGF-*β*RI/II inhibitor LY2109761 was used to block TGF-*β*/Smad signaling pathway; then JPJD was added. The results showed that, under this condition, JPJD had little effect on Snail and E-cadherin expression (Figure 4(a), Supplementary Figure 1).

Since TGF-*β*-induced Smad phosphorylation occurred within 60 min after TGF-*β* stimulation, we further examined the effect of JPJD on TGF-*β*-induced Smad phosphorylation and nuclear translocation after 60 min of TGF-*β* stimulation. The results in Figure 4(b) and Supplementary Figure 4 demonstrated that, after 60 min of TGF-*β* stimulation, the Smad phosphorylation increased obviously, as well as the increased accumulation of p-Smad 2/3 in the nuclear part of TGF-*β*-stimulated LoVo cells. However, the TGF*β*RI/II inhibitor (LY2109761) and JPJD reversed the former process. Being pretreated with LY2109761, the TGF-*β*-stimulated cells were again treated with JPJD, and the results showed no more inhibitory effect of JPJD on Smad phosphorylation and nuclear translocation, which indirectly demonstrated the inhibitory effect of JPJD on the activation of TGF-*β*-receptor. In addition, to enhance the convincingness of the above results, we have also used the previous constructed overexpressing vectors pcDNA3.1-Snail [10] and found that reexpression of Snail could partly recover the process of EMT that had been inhibited by JPJD (Figure 4(c), Supplementary Figure 5). All the above data suggested that JPJD might regulate the expression of Snail and E-cadherin through TGF-*β*/Smads signaling pathway, thereby inhibiting TGF-*β*-induced EMT, which might be one of the mechanisms through which JPJD inhibited the CRC invasion and metastasis.

Considering that JPJD has strong inhibitory effect on TGF-*β*-induced EMT, the underlying possible mechanism is very essential to explore. Real-time PCR was performed to detect the mRNA expression of E-cadherin and Snail which were closely associated with EMT. TGF-*β* stimulation made a significant increase in the mRNA expression of Snail, while the transcription level of E-cadherin was significantly downregulated, suggesting that TGF-*β* induced the EMT of LoVo cells through upregulating Snail transcription and downregulating E-cadherin transcription. Under the treatment of JPJD, the mRNA expression of Snail is downregulated, while the E-cadherin mRNA showed increased expression in a concentration-dependent manner (Figure 4(d)). This study suggested that JPJD inhibited the TGF-*β*-stimulated EMT by inhibiting the mRNA expression of Snail and upregulating the mRNA expression of E-cadherin.

The promoter activity of CDH1 gene encoding E-cadherin was measured by dual-luciferase assay. After stimulation by TGF-*β* for 48 h, the promoter activity of CDH1 in LoVo cells increased significantly, but when the JPJD was added simultaneously, the promoter activity of CDH1 decreased in a concentration-dependent manner (Figure 4(e)). The results demonstrated that JPJD inhibited the mRNA expression of E-cadherin by suppressing the promoter activity of CDH1 gene.

### 3.4. Inhibitory Effect of JPJD on the Metastasis of CRC Cells in Nude Mice

**Figure 5:**
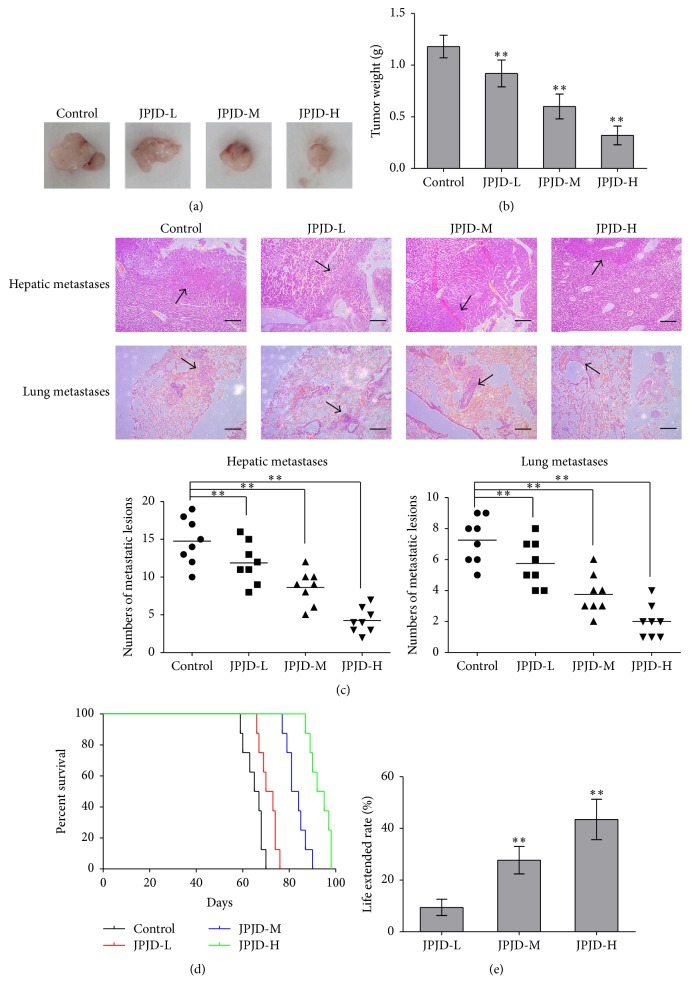
Inhibitory effect of JPJD on the metastasis of CRC cells in nude mice. (a, b) LoVo cells were used to establish the model of orthotopic transplantation tumor. After two weeks, the mice were randomized into four groups of 8 animals each. JPJDs with different doses were intragastrically administrated every day for the above four groups. After 28 days, the nude mice were sacrificed. The primary tumors were photographed and determined for tumor weights. (c) HE staining was applied to detect the liver and lung metastases in tumor-bearing mice, and the numbers of metastatic lesions in each group were determined. (d) Effect of JPJD on the survival time of tumor-bearing mice. (e) Effect of JPJD on the life extended rate of tumor-bearing mice. Here, JPJD-L is JPJD low-dose (250 mg/kg/day), JPJD-M is JPJD middle-dose (500 mg/kg/day), and JPJD-H is JPJD high-dose (1000 mg/kg/day). ^*∗*^*P* < 0.05 and ^*∗∗*^*P* < 0.01, versus control mice; ^#^*P* < 0.05  ^##^*P* < 0.01, versus JPJD-L group.

In order to investigate whether JPJD can inhibit the metastasis of orthotopic CRC tumor in nude mice, HE staining was applied to detect the liver and lung metastases in tumor-bearing mice. Firstly, LoVo cells were used to establish the model of orthotopic transplantation tumor, and the mice models were randomized into four groups. JPJDs with different doses were intragastrically administrated every day for the above four groups. After 28 days, the nude mice were sacrificed, and the primary tumors as well as the lung and liver of the mice were surgically removed. The images of orthotopic tumors showed that JPJD could inhibit the growth of tumors in a dose-dependent manner (Figure 5(a)), and the tumor weights also supported the former results (Figure 5(b)). Furthermore, by HE staining, a large number of metastases were found in the liver and lung tissues of orthotopic CRC tumor in nude mice. However, in the JPJD group, whether the low-dose or the middle- and high-dose, the metastases in the liver and lung tissues were fewer. Especially in the high-dose group, few metastases in the liver and lung tissues were found (Figure 5(c)). These data suggested that the traditional Chinese medicine JPJD can significantly inhibit the liver and lung metastasis of orthotopic CRC tumor in nude mice. We also observed the effect of JPJD on the survival time of tumor-bearing mice and found that the average survival times of the nude mice in the control group, low-dose JPJD group, middle-dose JPJD group, and high-dose JPJD group were 65.0, 71.1, 83.0, and 93.2 days, respectively (Figure 5(d)). JPJD of low-dose, middle-dose, and high-dose prolonged the mice life by 9.42%, 27.69%, and 43.46 (Figure 5(e)). The above results showed that JPJD can significantly prolong the survival time of tumor-bearing in a dose-dependent manner.

### 3.5. JPJD Inhibited EMT In Vivo through TGF-*β*/Smad Signaling Pathway

**Figure 6:**
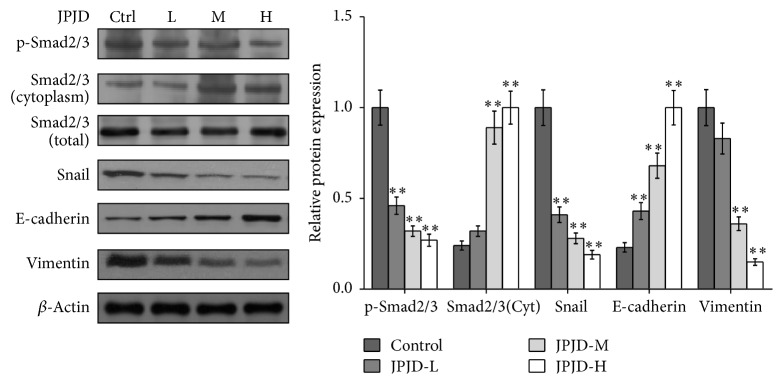
JPJD inhibited the EMT in vivo through TGF-*β*/Smad signaling pathway. Western blot was performed to detect the effect of JPJD on the process of EMT, as well as the levels of p-Smad2/3, Smad2/3, and Snail in the orthotopic CRC tumor tissues. Here, JPJD-L is 250 mg/kg/day, JPJD-M is 500 mg/kg/day, and JPJD-H is 1000 mg/kg/day. ^*∗*^*P* < 0.05 and ^*∗∗*^*P* < 0.01, versus LoVo cells without JPJD treatment.

In the in vivo experiment, the data showed that JPJD can upregulate the expression of E-cadherin and downregulate the expression of Vimentin in the orthotopic CRC tumor tissues (Figure 6, Supplementary Figure 6), which also showed the significance of JPJD on inhibiting EMT in vivo. Moreover, JPJD can downregulate the total levels of p-Smad2/3 and Snail and upregulate the levels of Smad2/3 in the cytoplasm but have little effect on the total levels of Smad2/3 in the orthotopic CRC tumor tissues (Figure 6, Supplementary Figure 6). These data further implied that JPJD might inhibit EMT through regulating the TGF-*β*/Smads signaling pathway, as well as the expression of Snail and E-cadherin.

## 4. Discussion

The mortality of CRC in the United States ranked third in malignant tumors, and in China the incidence and mortality of CRC are both increasing year by year [1]. EMT often occurs in the progression of various epithelial cell carcinomas, and it is closely associated with invasion and metastasis of numerous tumors [2, 3]. Studies have shown that, in the process of EMT, the epithelial cells lose their polarity, reduce their contact with the surrounding cells and matrix, and decrease the adhesion and interactions between cells, and the abilities of cells migration and movement enhance. In addition, the cells lose their epithelial phenotype, accompanied by a reduction of E-cadherin expression, and gradually gain mesenchymal phenotype, along with an increase of Vimentin expression. In in vitro experiments, cytokines such as TGF-*β*, HGF, and EGF are generally used to induce EMT [4–6], and, in our study, TGF-*β* was selected to perform the stimulating function.

JPJD is a traditional Chinese medicine compound from clinical experience and is mainly used for the prevention of gastrointestinal cancer recurrence and metastasis [7]. A variety of basic researches have proved the effective antitumor function of JPJD [8, 9]. In this study, we have shown that JPJD had the inhibitory effect on TGF-*β*-induced EMT in CRC cells, and we have also explored the underlying mechanism.

Tirino et al. found that, after TGF-*β* stimulation, the A549 cells showed the EMT performance, and the cells invasion and metastasis significantly enhanced [11]. Similarly, Gao et al. also found that TGF-*β* isoforms could induce EMT and promote metastasis of ovarian cancer cells [12]. In respect to our research, we found that TGF-*β* could induce the EMT of CRC cells and enhance the invasion and metastasis of CRC cells. Since the invasion and metastasis ability of EMT-transited CRC cells enhanced, to study the impact of traditional Chinese medicine on the EMT becomes very meaningful. There have been numerous reports about Chinese medicine monomer inhibiting EMT. For example, Liu et al. found that berberine can inhibit metastasis of prostate cancer cells by inhibiting the expression of EMT related genes [13]. Vergara et al. found that resveratrol can inhibit EGF-induced EMT in breast cancer MCF-7 [14]. Comparatively speaking, studies about traditional Chinese medicine compound inhibiting EMT process in cancer are very few. Zhang et al. found that QingYin HuaJi Recipe can inhibit the invasion and metastasis of pancreatic cancer cells by inhibiting EMT [15]. In order to investigate the effect of JPJD on TGF-*β*-induced EMT in CRC cells, we chose the JPJD of low concentration regarding negligible cytotoxicity, to observe its effect on the process of EMT. The data demonstrated that JPJD could inhibit TGF-*β*-induced EMT, as well as the invasion and metastasis in a concentration-dependent manner. Moreover, the in vivo results demonstrated that JPJD can significantly inhibit the liver and lung metastasis of orthotopic CRC tumor in nude mice, as well as significantly prolonging the survival time of tumor-bearing in a dose-dependent manner.

The occurrence of EMT involved the regulation of many genes, and several studies have implied the importance of E-cadherin and Snail in regulating EMT [16, 17], and these EMT-associated transcription factors are the potential targets for traditional Chinese medicine. Further studies have shown that JPJD inhibited TGF-*β*-induced EMT by downregulating the mRNA expression of Snail and upregulating the mRNA expression of E-cadherin, and JPJD inhibited the mRNA expression of E-cadherin by suppressing the promoter activity of CDH1 gene, which encoded E-cadherin protein. In vivo, we further demonstrated that JPJD can inhibit EMT through regulating the TGF-*β*/Smads signaling pathway and the expression of Snail and E-cadherin.

TGF-*β*/Smad signaling pathway is closely associated with the proliferation, differentiation, migration, and so on. TGF-*β* could bind and phosphorylate cell-surface receptors (TGF-*β*RI/TGF-*β*RII), and the activated TGF-*β*RI phosphorylates Smad2 or Smad3 will subsequently bind to Smad4 [18, 19]. The Smad complex moves into the nucleus and interacts with various transcription factors to regulate the transcription of downstream genes [20–22]. Snail was one of the mediated genes by TGF-*β*/Smad signaling pathway [10, 23, 24]. In our study, TGF-*β*/Smad signaling pathway can repress the E-cadherin expression, promote the EMT process, and finally elevate the ability of CRC cells invasion and metastasis. Nevertheless, JPJD could inhibit the invasion and metastasis by reducing the transcriptional activities of EMT-associated factors Snail and E-cadherin during the initiation of TGF-*β*/Smad-induced EMT.

In summary, our new findings provided evidence that JPJD could inhibit TGF-*β*-induced EMT in CRC through TGF-*β*/Smad2/3 mediated Snail/E-cadherin expression, and this might be the potential effect mechanism of JPJD on inhibiting the invasion and metastases of CRC in vitro and in vivo. All the results provide a reliable experimental basis for targeted therapy of CRC.

## Supplementary Material

Supplementary Figure 1 All the original western blot figures from three repeated experiments were presented here, corresponding to Figure 1(b) and Figure 1(c). Supplementary Figure 2 All the original western blot figures from three repeated experiments were presented here, corresponding to Figure 3(d). Supplementary Figure 3 All the original western blot figures from three repeated experiments were presented here, corresponding to Figure 4(a). Supplementary Figure 4 All the original western blot figures from three repeated experiments were presented here, corresponding to Figure 4(b). Supplementary Figure 5 All the original western blot figures from three repeated experiments were presented here, corresponding to Figure 4(c). Supplementary Figure 6 All the original western blot figures from three repeated experiments were presented here, corresponding to Figure 6.
