# Supplementary material for: JianPi JieDu Recipe Inhibits Epithelial-to-Mesenchymal Transition in Colorectal Cancer through TGF-β/Smad Mediated Snail/E-Cadherin Expression
Source: Biomed Res Int. 2017 Feb 16;2017:2613198. doi: 10.1155/2017/2613198 (PMC5337333; doi:10.1155/2017/2613198)
Supplement: Supplementary file 1 — Supplementary Figure 1 All the original western blot figures from three repeated experiments were presented here, corresponding to Figure 1(b) and Figure 1(c). Supplementary Figure 2 All the original western blot figures from three repeated experiments were presented here, corresponding to Figure 3(d). Supplementary Figure 3 All the original western blot figures from three repeated experiments were presented here, corresponding to Figure 4(a). Supplementary Figure 4 All the original western blot figures from three repeated experiments were presented here, corresponding to Figure 4(b). Supplementary Figure 5 All the original western blot figures from three repeated experiments were presented here, corresponding to Figure 4(c). Supplementary Figure 6 All the original western blot figures from three repeated experiments were presented here, corresponding to Figure 6. [file 2613198.f1.pptx]

## Slide 1
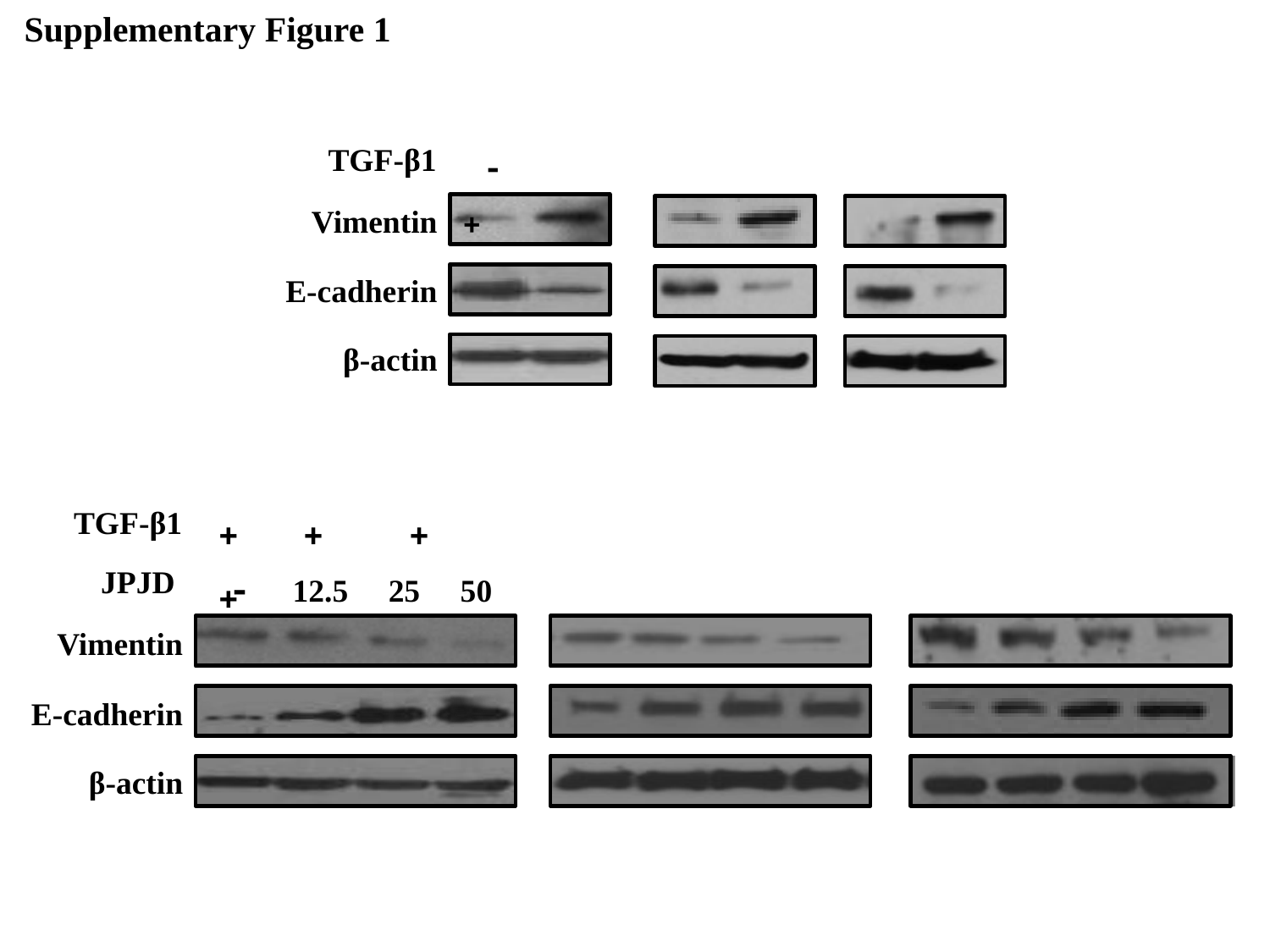

Supplementary Figure 1
 - +
TGF-β1
 Vimentin
E-cadherin
β-actin
+ + + +
TGF-β1
 - 12.5 25 50
JPJD
 Vimentin
E-cadherin
β-actin

## Slide 2
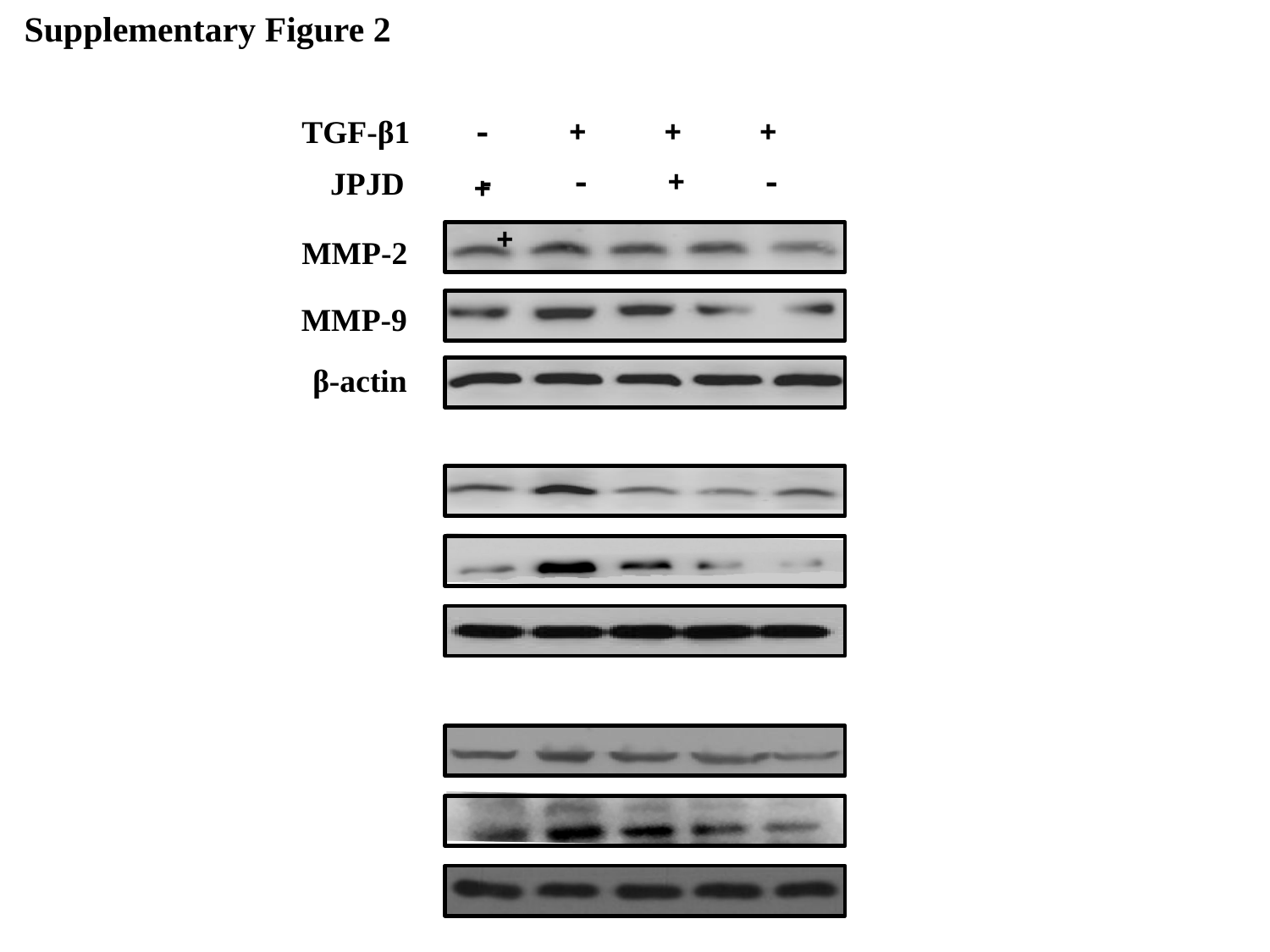

Supplementary Figure 2
- + + + +
TGF-β1
 - - + - +
JPJD
MMP-2
MMP-9
β-actin

## Slide 3
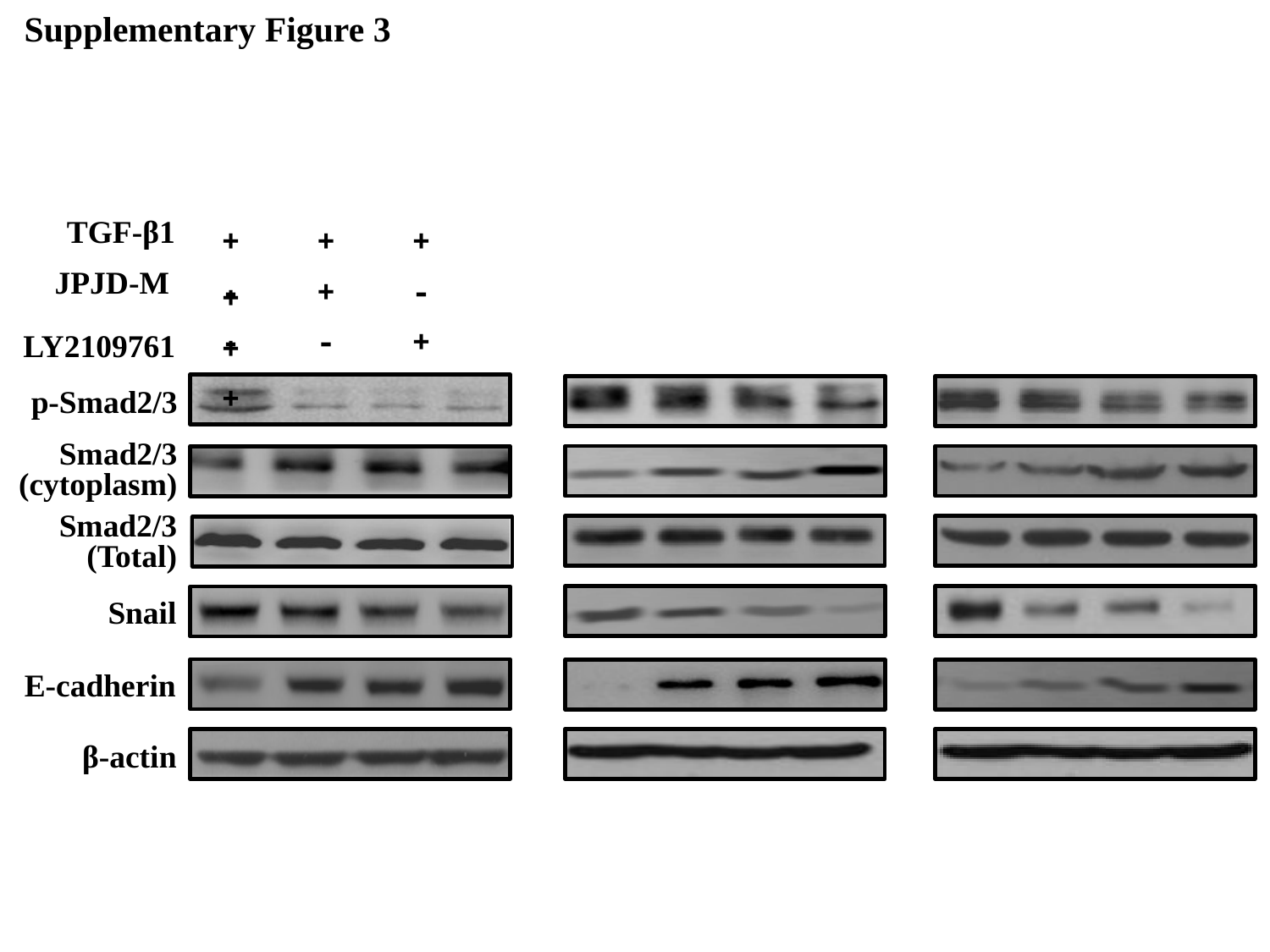

Supplementary Figure 3
TGF-β1
LY2109761
 + + + +
 - + - +
JPJD-M
 - - + +
p-Smad2/3
Smad2/3
(cytoplasm)
Smad2/3
(Total)
Snail
E-cadherin
β-actin

## Slide 4
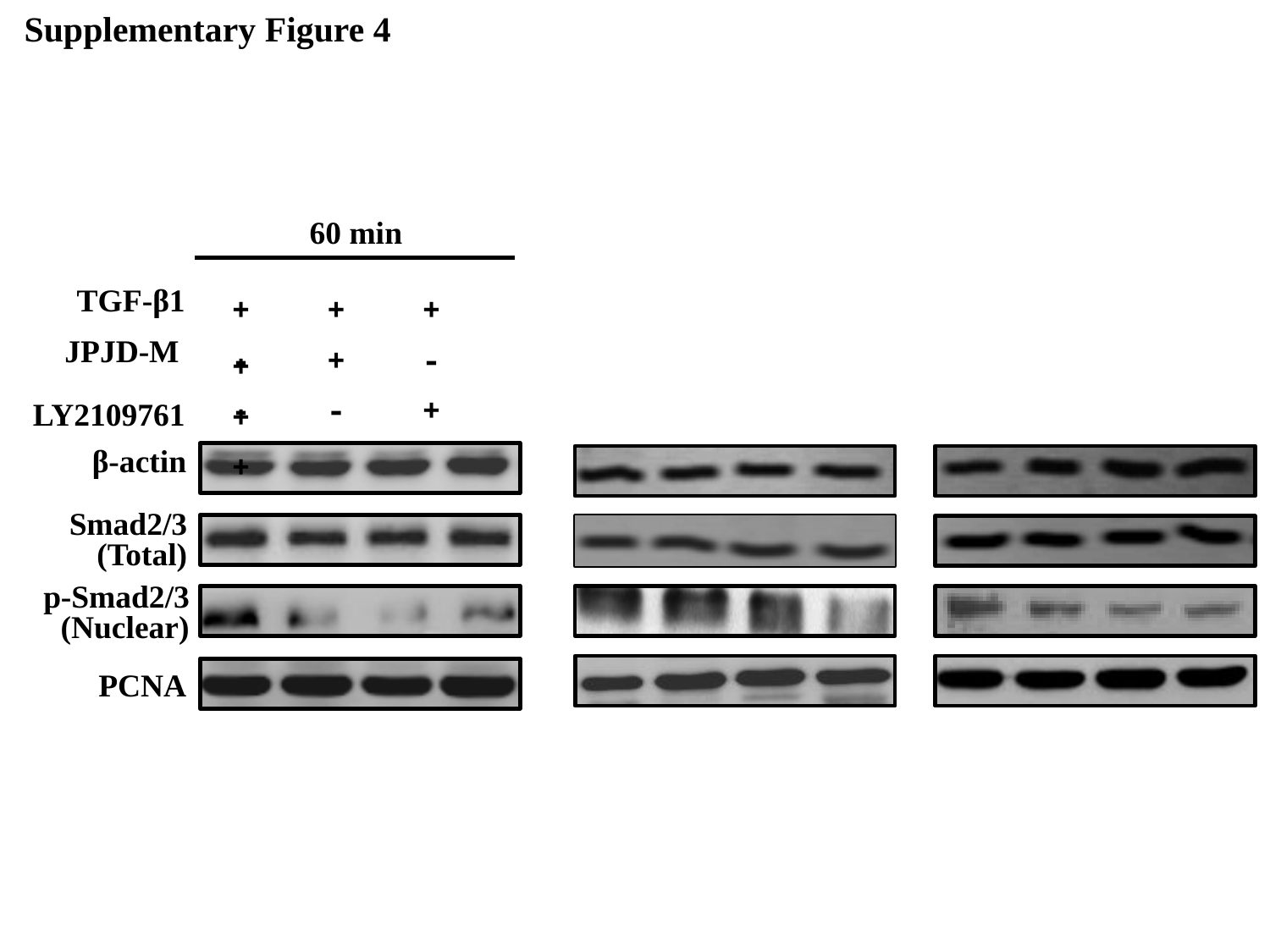

Supplementary Figure 4
60 min
TGF-β1
LY2109761
 + + + +
 - + - +
JPJD-M
 - - + +
β-actin
Smad2/3
(Total)
p-Smad2/3
(Nuclear)
PCNA

## Slide 5
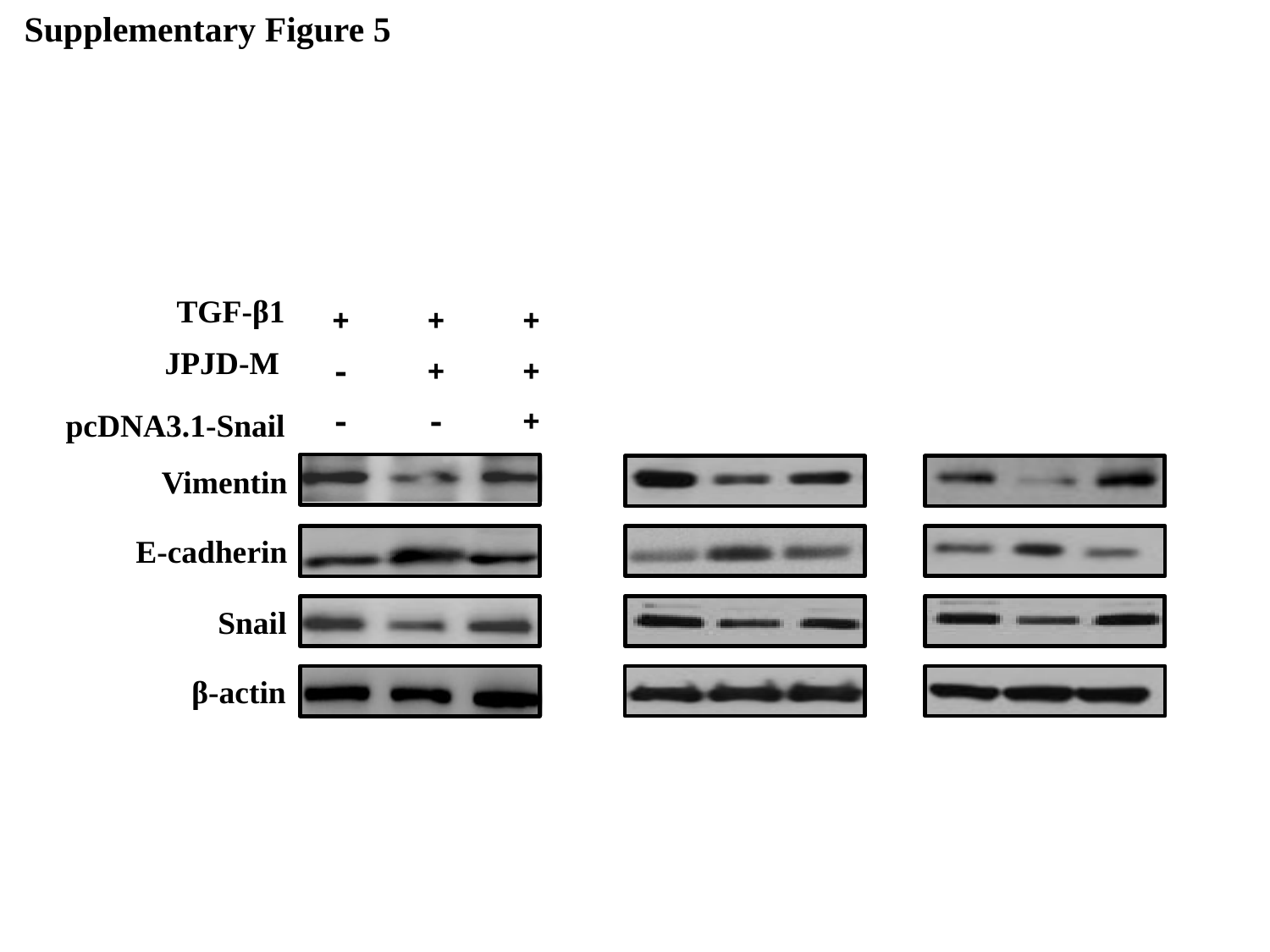

Supplementary Figure 5
TGF-β1
pcDNA3.1-Snail
 + + +
 - + +
JPJD-M
 - - +
Vimentin
E-cadherin
Snail
β-actin

## Slide 6
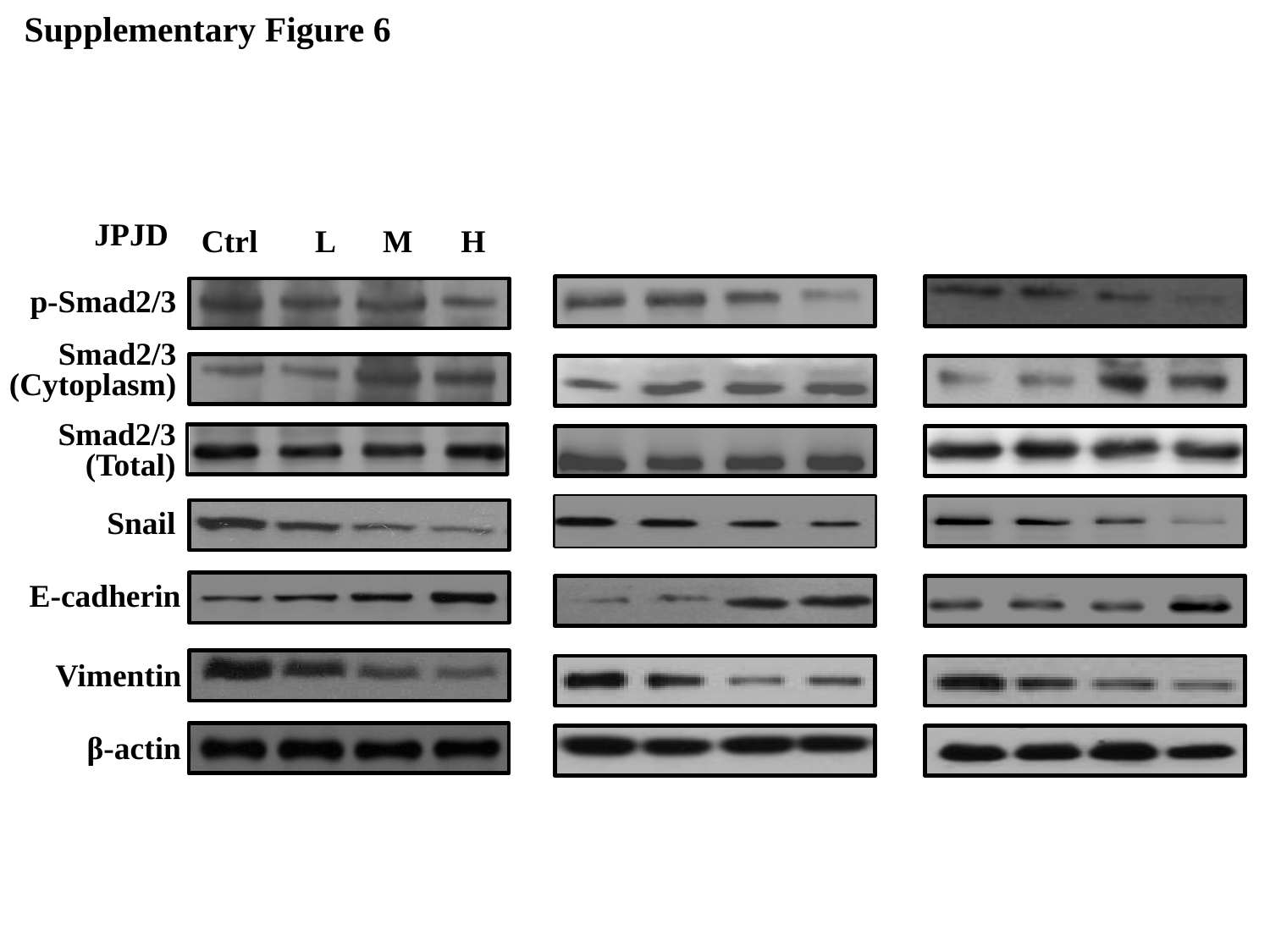

Supplementary Figure 6
Ctrl L M H
JPJD
p-Smad2/3
Smad2/3 (Cytoplasm)
Smad2/3 (Total)
Snail
E-cadherin
 Vimentin
β-actin
